# Evaluation on Analgesic and Anti-Inflammatory Activities of Total Flavonoids from* Juniperus sabina*

**DOI:** 10.1155/2018/7965306

**Published:** 2018-07-05

**Authors:** Jun Zhao, Aosimanjiang Maitituersun, Chenyang Li, Qian Li, Fang Xu, Tao Liu

**Affiliations:** ^1^Xinjiang Key Laboratory for Uighur Medicine, Institute of Materia Medica of Xinjiang, Urumqi 830004, China; ^2^Department of Toxicology, College of Public Health, Xinjiang Medical University, Urumqi 830011, China

## Abstract

The leaves of* Juniperus sabina* (Cupressaceae) are used in traditional Uygur medicine for the treatment of rheumatism and arthritic pain. This study aimed to investigate the analgesic and anti-inflammatory effects of total flavonoids from leaves of* Juniperus sabina* (JSTF) on rodents. The anti-inflammatory activity was investigated using the carrageenan, egg albumin, or histamine-induced rat paw edema as well as xylene-induced ear edema, capillary permeability, and cotton pellet granuloma while the antinociceptive activity was evaluated using the mouse writhing, formalin, and hot-plate tests. JSTF (125, 250, 500 mg/kg) significantly inhibited xylene-induced ear edema in mice (inhibition ratio as 16.22%, 40.67%, and 51.78%, respectively) and also significantly ameliorated acetic acid increased vascular permeability in mice (inhibition ratio as 11.63%, 32.56%, and 53.49%, respectively). JSTF (250 and 500 mg/kg) gave significant reduction of carrageenin-induced paw oedema at the interval of 1 h and 5 h. Administration of JSTF (500 mg/kg) caused a significant anti-inflammatory effect against oedema induced by egg albumin or histamine at the interval of 0.5 h and 4 h, and both which induced the paw oedema were also inhibited by JSTF (250 mg/kg) at a point in 1, 2, or 3 h after the inflammation. Furthermore, JSTF (125, 250, and 500 mg/kg) produced time-course increase in pain threshold in hot-plate test also. JSTF produced concentration-dependent inhibition on cyclooxygenase-2 (COX-2) or 5-lipoxygenase (5-LO) activities* in vitro*, and their IC_50_ values were 31.92 and 129.26 *μ*g/mL, respectively. Moreover, JSTF significantly caused a significant dose-dependent inhibition on acetic acid induced writhing response in mice (inhibition ratio as 23.27%, 36.91%, and 50.76%, respectively). JSTF also significantly ameliorated formalin-induced pain in mice in the late phase on dose-dependent way. These results confirms the clinical use of* J. sabina* for treatment of rheumatoid arthritis in ethnomedicine, and its effective mechanism will be further studied in the future.

## 1. Introduction

The plant in Juniper genus with around 70 species is widely distributed in temperate regions in Asia, and these species have been used to various inflammatory diseases such as bronchitis, rheumatism, fungal infections, hemorrhoids, and gynecological diseases in many countries.* Juniperus sabina*, also known as “Chou Juniper”, “Xinjiang Juniper”, or “Andorra Creeping Juniper”, is produced in arid or semiarid sand as well as the mountain slope in temperate regions [1, 2]. As a traditional ethnic medicine,* J. sabina* leaves have the efficacies of flooding wind calm, invigorating blood circulation and alleviating pain and have been used to treat rheumatism and arthritic pain in China for a long history [3, 4]. The extracts from this plant have been reported as antimicrobial, antioxidant, antitumor, antifertility, and anti-inflammatory activities [5]. Chemical constituents of this plant include essential oil, terpene, lignans, flavonoids, and phenolic acid, of which flavonoids were mainly characteristic constituents and its contents is 3.12% [6, 7]. In the previous study, effect of total flavonoids from* J. sabina* (JSTF) on adjuvant-induced arthritis (AA) in rats was investigated, and the result showed that JSTF had significantly preventive effects on AA in rats [8]. On the basis of the above research, this study aims to systematically evaluate analgesic and anti-inflammatory effects of JSTF by various animal models and provide basic data for its further development and utilization.

## 2. Materials and Methods

### 2.1. Drugs, Chemicals, and Apparatus

The drugs and chemicals used in the study were Aspirin Enteric-Coated Tablets (Bayer, German), cyclooxygenase-2/5-lipoxygenase (COX-2/5-LO) colorimetric inhibitor screening assay kit (Cayman, USA), histamine (Sinopharm Chemical Reagent, China), Evans blue (Fortuneibo-tech, China), and carrageenin (Shanghai Beilian Biotech, China). Other chemicals used in these experiments were of analytical grade from commercial sources.

### 2.2. Collection of Plant Material

*J. sabina* leaves were collected from the southern mountain at Urumqi of Xinjiang in China, in July 31, 2015. The plant material was authenticated by associate researcher Jiang He in Xinjiang Institute of Materia Medica. A voucher specimen (XJYSZH-02) was deposited in Xinjiang Institute of Materia Medica in China.

### 2.3. *Preparation of *Total Flavonoids from* J. sabina* (JSTF)

The powdered leaves of* J. sabina* (10.0 kg) were defatted at reflux condition with petroleum ether and extracted under reflux at 80°C with 70% ethanol for 1 h in three batches to yield a dark brown residue (2.16 kg). After being dissolved in water, the extract was purified by D101 adsorption macroporous resin and polyamide resin to obtain total flavonoids (JSTF, 420 g).

Total flavonoids content in JSTF was determined according to described methods in Chinese pharmacopeia [9]. Approximately 1.0 mL samples or standard solutions were mixed with 1.0 mL of 5% NaNO_2_ solution. After 6 min, 1.0 mL 10% Al(NO_3_)_3_ solution was added, and the mixture was allowed to stand for another 6 min. Subsequently, the reaction solution was mixed with 10.0 mL 4% NaOH solution and deionized water was immediately supplied to obtain a final volume of 25 mL, followed by thorough mixing and standing for another 10 min. Absorbance of the mixture was determined at 509 nm, and total flavonoid contents were calculated as rutin according to a calibration curve (y = 11.249x + 0 0044, R^2^ = 0 9999, linearity range at 0.2–1.8 mg of rutin). Data were presented as the average of triplicate analyses.

### 2.4. HPLC Analysis of JSTF

High performance liquid chromatography (HPLC) method was developed for simultaneous quantification of three major flavonoids (rutin, quercitrin, and isoquercitrin) in JSTF. Phenomenex Gemini-NX C18 column (250 mm × 4.6 mm, 5 *μ*m, Milford, USA) was used for all the analyses. The mobile phase composed of A (acetonitrile) and B (0.2% phosphoric acid, v/v) with a gradient elution: 0–2 min, 19-20%A; 2–20 min, 20–24%A; 20–25 min, 24–19%A. The flow rate of the mobile phase was 1.0 mL/min and the column, autosampler temperatures were maintained at 30°C, and detection wavelength was maintained at 360 nm. The contents of rutin, quercitrin, and isoquercitrin were simultaneously determined, respectively, under this chromatographic condition, and their contents were calculated according to calibration curve (y = 15872905.4945x + 4299.8370, R^2^ = 0 9999, linearity range at 10.4-62.4 *μ*g/mL; y = 18830685.1984x + 5815.6423, R^2^ = 0 9999, linearity range at 11.0-66.0 *μ*g/mL; y = 21849554.2857x + 2658.8777, R^2^ = 0 9999, linearity range at 3.0-18.0*μ*g/mL, respectively). Data were presented as the average of triplicate analyses.

### 2.5. COX-2/5-LO Inhibition Assay

To investigate whether the anti-inflammatory activity of JSTF may be attributed in part to an inhibition of arachidonic acid (AA) mediated inflammatory effects, the COX-2 and 5-LO inhibitory effects of JSTF were assayed according to the method described in the assay kits, respectively. The tests were divided seven groups as follows: blank group, control group, and JSTF groups (the final concentrations of 12.5, 25, 50, 100, or 200 *μ*g/mL). In COX-2 inhibitor screening experiment, 150 *μ*L assay buffer (0.1M Tris-HCl, PH=8), 10 *μ*L heme 10 *μ*L, and COX-2 enzyme were successively added to inhibitor wells and control wells. 10 *μ*L JSTF solutions were added to inhibitors wells. 10 *μ*L water was used to the control wells and blank wells (160 *μ*L of assay buffer plus 10 *μ*L of heme), respectively. After being incubated for five minutes at 25°C, 20 *μ*L of the colorimetric substrate solution and 20 *μ*L of arachidonic acid (AA) were added to all the wells. The absorbance (A) was read at 590 nm after shaking the plate for 20 s and incubated for two minutes at 25°C, In 5-LO inhibitor screening experiment, 10 *μ*L of JSTF solution and 10 *μ*L water were added to inhibitor wells (90 *μ*L of 5-LO) and blank wells (100 *μ*L of assay buffer) simultaneously. After being incubated for 5 min at 25°C, 10 *μ*L AA was added and initiating the reaction more than 10 min. At last, 100 *μ*L chromogen was added for stopping enzyme reaction, covering with a plate cover, placing the 96-well plate on a shaker for 5 min, and reading the absorbance at 490 nm. Each experiment was done in parallel three times. Inhibition (%) = (OD_control_-OD_sample_) /(OD_control_ - OD_blank_) × 100%.

### 2.6. Animals

Sprague-Dawley rats (200.0 ± 20.0 g) and Kunming mice (20.0 ± 2.0 g) were supplied by the Experimental Animal Centre of Xinjiang Medical University in China (No. SYXK(xin) 2011-0004). Animals in various models were used according to the principle of half male and half female except for only female mice in hot-plate test. The animals were allowed free access to food and water under a 12h/12h light/dark cycle with the room temperature maintained at 25 ± 1°C and relative humidity of 40-60%. In anti-inflammatory and analgesic experiments, animals were randomly divided into five groups: control, aspirin (200 mg/kg), and JSTF (125, 250, and 500 mg/kg). The study protocols were approved by the Ethics Committee on Animal Experiment, Xinjiang Material Medica, China.

### 2.7. Acute Toxicity Assay

Mice were generally fasted for 12 h before the test [10]. Six groups of mice (n=10) received 10 mL/kg distilled water and doses (14, 16, 18, 22, and 24  mg/kg, p.o.) of JSTF dissolved in distilled water. The animals were observed continuously for first 72 hours and 14 days for their general behavioral symptoms: body weight changes, hazardous symptoms and mortality body weight, toxicity, behavioral, and viscera changes. On 15th day, mice were killed, and all organs and tissues were observed macroscopically.

### 2.8. Anti-Inflammatory Activities

#### 2.8.1. Xylene-Induced Ear Edema

This experimental procedure was performed using the method of Hosseinzadeh* et al. *[11]. Mice were used, and test drugs were administered orally on a once daily dosage regimen for 5 days, and the control group received vehicle. The oedema was induced in each mouse by applying 50 *μ*L xylene to the inner surface of the right ear. Ninety minutes after xylene daubing, the mice were executed by cervical dislocation, and both ears were removed and weighed [12]. The difference between the right and left ears was determined for each group, and % inhibition = (Difference of ear weight in control group - Difference of ear weight in test group)/Difference of ear weight in control group ×100.

#### 2.8.2. Acetic Acid-Induced Vascular Permeability

This test procedure was performed according to the described method [13] with a slight modification. Mice were used, and test drugs were administered orally for 7 days. 1 h after the last treatment, mice were intraperitoneally injected with 0.6% acetic acid in saline solution (10 mL/kg). Whereafter 0.5% Evans blue solution (10 mL/kg) in normal saline was injected intravenously through tail vein. Twenty minutes later, mice were sacrificed by cervical dislocation and the peritoneal cavity was washed with normal saline (5 mL). The washings were collected and made volume up to 10 mL into heparinized test tubes and subsequently centrifuged at 3,000 rpm for 15 min. The dye content in the supernatant was measured spectrophotometrically at a wavelength of 590 nm. The vascular permeability effects were expressed as the total amount of dye leaked into the intraperitoneal cavity based on their absorbance, and inhibition% was determined using the equation: (Mean absorbance in control-Mean absorbance in test) ×100/Mean absorbance in control.

#### 2.8.3. Carrageenan-Induced Rat Paw Edema

This test was performed according to the reported methods [12]. Rats were used, and test drugs were administered orally for 5 days. One hour after the last treatment, the oedema was induced by injection of 100 *μ*L carrageenan (1% in normal saline) in the subplantar tissue of the right hind paw. Paw volume was measured using the toe volume measure meter before and 1, 2, 3, 4, and 5 h after injection of carrageenan. Results were expressed as the increase in paw volume (in ml) calculated after subtraction of basal paw volume.

#### 2.8.4. Egg Albumin-Induced Inflammation

This test was performed according to the reported method [14]. Inflammation was induced in rats by the injection of egg albumin (0.1 mL, 20% in normal saline) into the subplantar tissue of the right hind paw. Test drugs were administered to 24 h fasted rats 1 h before the induction of inflammation. The swelling degree of the injected paw was measured before and 0.5, 1, 2, 3, and 4 h after the administration of the phlogistic agent. Results were expressed as the increase in paw volume (in ml) calculated after subtraction of basal paw volume.

#### 2.8.5. Histamine-Induced Inflammation

This test was performed according to the reported method [15]. Inflammation was induced in mice by the injection of 0.1 mL 0.2% histamine in normal saline into the subplantar tissue of the right hind paw in rats. Test drugs were administered to rats at 1 h before the induction of inflammation. Control group received 10 mL/kg of distilled water orally. Edema was assessed as the difference in paw volume between the control and 0.5, 1, 2, 3, and 4 h after the administration of the inflammatory agent, inhibition.

#### 2.8.6. Cotton Pellet Induced Granuloma

This test was performed as described by Gupta* et al.* [16]. Sterilized cotton pellets of 40 ± 1 mg weight each were implanted subcutaneously into the rat's groin under sterile technique. Soon afterwards, test drugs were administered orally for seven consecutive days. The rats were executed by cervical dislocation on the eighth day. The cotton pellets were removed surgically from extraneous tissues. The dry cotton weight was recorded after dried at 60°C for 24 h. The weight difference between dry cotton and the cotton before implantation is considered as weight of granuloma formed. Inhibition% was calculated using the following equation: (Weight of pellet in control group - Weight of pellet in test group)/Weight of pellet in control group×100.

### 2.9. Analgesic Effect of JSTF

#### 2.9.1. Acetic Acid Induced Writhing

This test was performed according to the reported method [17]. Sixty minutes before an intraperitoneal injection of 0.6% acetic acid solution (10 mL/kg body weight), each mouse was intragastrically administered with a test drug or vehicle. The number of writhing responses was recorded for each animal during a subsequent 10 min period after the administration of acetic acid. The percentage inhibition of writhing was calculated using the following formula: Inhibition (%) = (Number of writhing in control-Number of writhing in test)/Number of writhing in control ×100.

#### 2.9.2. Hot-Plate Test

This test was performed according to the method described by Meng* et al.* [18]. Each female mouse was kept on a hot plate having a constant temperature of 55 ± 5°C, and response time was recorded as the time elapsed before the mouse responded by licking of a hind paw. Only mice with a control response of 10~30 sec were included in the study. The nociceptive response was measured 30 min after treatment and every 30 min for 2 h. The increase in latency time in relation to the initial time for each group was taken as an index of analgesic activity.

#### 2.9.3. Formalin Test

This procedure was essentially similar to that described by Ishola* et al.* [19]. Test drugs were administered orally on a once daily dosage regimen for 5 days, and the control group received vehicle. One hour after the last administration, 10 *μ*L of formalin (1.8 mol/L in saline) was injected into the right hind paw of each mouse. The time (in sec) spent in licking or biting the injected paw, indicative of pain, was recorded for each animal. The responses of the mice were observed for the first 3 min (neurogenic phase) and 20~40 min (inflammatory phase) postformalin injection.

### 2.10. Statistical Analysis

Data were expressed as the mean values ± standard deviation (S.D.) for each measurement. The data were also analyzed by one-way analysis of variance (one-way ANOVA). Results with* P* < 0.05 were considered significant.

## 3. Results

### 3.1. Chemical Analysis

The content of flavonoids in the JSTF was determined quantitatively as 62.02 ± 0.76 g/100g, and the content of rutin, isoquercitrin, and quercitrin in JSTF were detected by HPLC to be 26.21 ± 0.80 mg/g, 8.42 ± 1.62 mg/g, and 22.48 ± 0.95 mg/g, respectively.

### 3.2. Acute Toxicity

Oral administration of highest dose 24.0 g/kg of JSTF did not showed any acute toxic symptoms, and no deaths occurred in the experiment. There were no significant differences in body weights and physiological or behavioral responses between the JSTF treated and control group, and there were also no changes in food or water intake. This result indicated that the treatment of JSTF was safe under the maximum dose at 24 g JSTF/kg body.

### 3.3. Inhibition Effects of JSTF on COX-2/5-LO In Vitro

**Figure 1:**
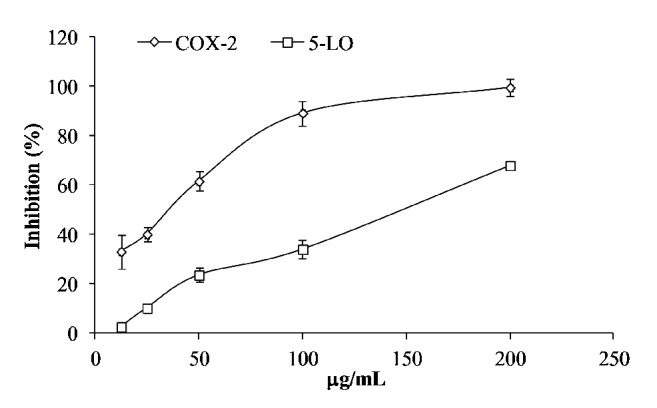
Inhibition effects of JSTF on COX-2/5-LO* in vitro* (mean ± S.D., n=3).

As shown in Figure 1, the activities of COX-2 and 5-LO were significantly inhibited by JSTF (IC_50_, 31.92 *μ*g/mL, 129.26 *μ*g/mL, respectively).

### 3.4. Anti-Inflammatory Activities

**Figure 2:**
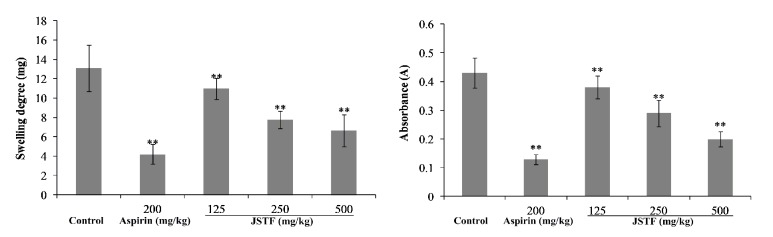
Effects of JSTF on the xylene-induced ear swelling and peritoneal capillary permeability in mice. Mean ± S.D., n=10; *∗∗P*<0.01, compared with control group.

Figure 2 represents effects of JSTF with xylene-induced ear edema and peritoneal capillary permeability model. JSTF (125, 250, and 500 mg/kg) significantly inhibited xylene-induced ear edema in mice in a dose-dependent manner (inhibition ratio showed as 16.22%, 40.67%, and 51.78%, respectively,* P*<0.01). JSTF (125, 250, 500 mg/kg) also significantly ameliorated acetic acid increased vascular permeability in mice (*P* < 0.01), and their inhibition ratio was calculated as 11.63%, 32.56%, and 53.49%.

**Figure 3:**
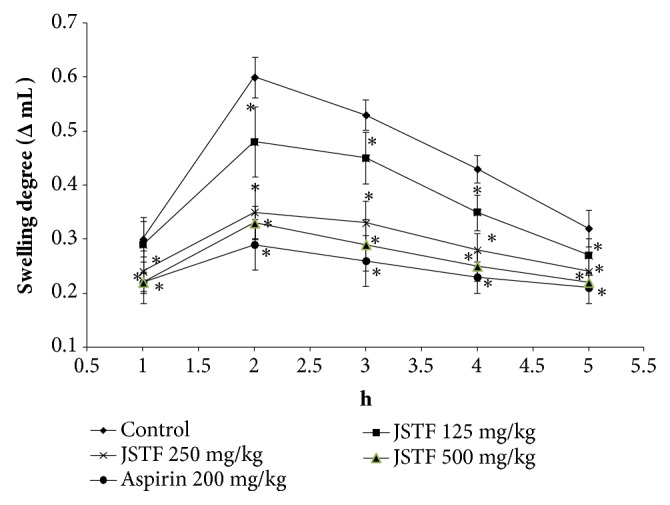
Effects of JSTF on the carrageenan-induced paw edema in rats. Mean ± S.D., n=10; *∗P*<0.05, compared with normal control group.

**Figure 4:**
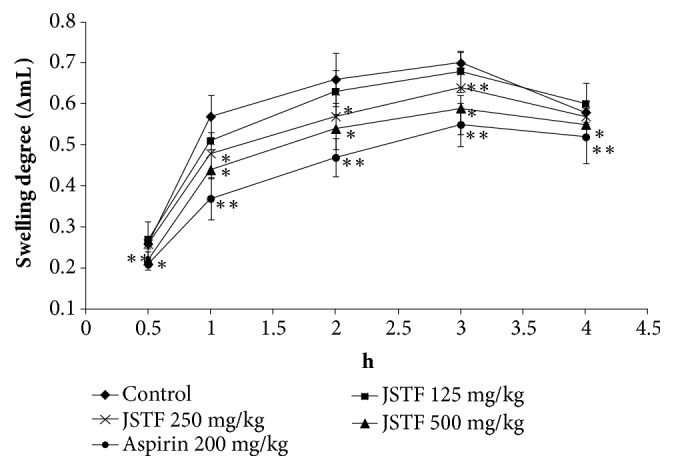
Effects of JSTF on egg albumin-induced paw edema in rats. Mean ± S.D., n=6; *∗P*<0.05,*∗∗P*<0.01, compared with normal control group.

**Figure 5:**
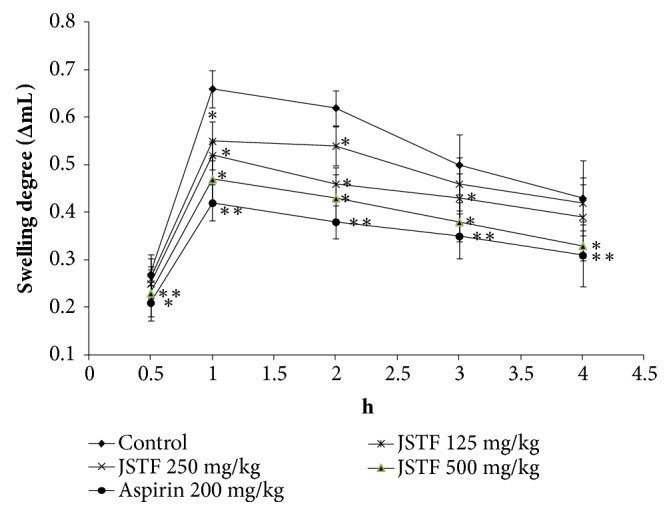
Effect of JSTF on histamine-induced paw edema in rats. Mean ± S.D., n=6; *∗P*<0.05,*∗∗P*<0.01, compared with normal control group.

The effect of JSTF on carrageenan-induced oedema was shown in Figure 3. Middle and high doses group of JSTF (250 and 500 mg/kg) gave significant reduction of carrageenin-induced paw oedema at the interval of 1 h and 5 h (*P* < 0.01). JSTF at the dose of 125 mg/kg also reduced carrageenin-induced paw oedema at the interval of 2 h and 5 h (*P* < 0.05). Administration of JSTF (250 and 500 mg/kg) on egg albumin-induced oedema in mice caused a significant (*P*<0.05) dose-dependent anti-inflammatory effect against oedema caused by egg albumin at the interval of 1 h and 3 h (Figure 4). JSTF at the different doses (125, 250, and 500 mg/kg) gave significant reduction of histamine-induced paw oedema at the interval of 1 h and 3 h (*P* < 0.01,* P* < 0.05; Figure 5).

Table 1 presents the effects of different doses of JSTF on cotton pellet granuloma test and exhibits a significant dose-dependent inhibition of granuloma formation at the site of inflammation in animal model. Potent anti-inflammatory response were observed with JSTF at doses of 125, 250, and 500 mg/kg, and inhibition rates were 15.38%, 18.48%, and 22.02%, respectively.

### 3.5. Analgesic Activity of JSTF

**Figure 6:**
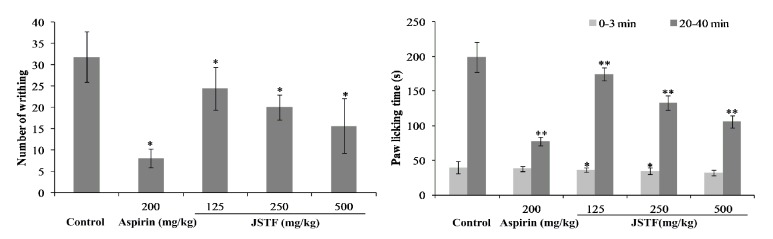
Effect of JSTF on acetic acid induced writhing response or formalin-induced licking response in mice Mean ± S.D., n=10; *∗P*<0.05, *∗∗P*<0.01, compared with control group.

To assess the analgesic activities of JSTF, we performed the acetic acid-induced abdominal writhing response and formalin-induced licking response in mice as well as hot-plate test. Acetic acid-induced abdominal writhing response is mainly based on the peripheral system, which involves prostaglandin synthesis via cyclooxygenase [20]. Administration of JSTF at different doses (125, 250, and 500 mg/kg) significantly decreased the number of writhing in mice (*P*<0.01) and the effect was found to be dose-dependent. The maximum inhibition of writhing was 50.76% at dose of 500 mg/kg of JSTF, and the effect was lower than aspirin (200 mg/kg, 74.61%). The results are given in Figure 6. Thus, JSTF possessed antinociceptive effect against the acetic acid-induced abdominal writhing response in mice.

We further found that JSTF significantly prevented the early and late phase of formalin-induced licking response in mice (Figure 2) (*P* < 0.05,* P* < 0.01). However, aspirin could only effectively inhibit the late but not early phase of formalin-induced licking response in mice (*P* < 0.01). In latency of response in the hot-plate method, mice pretreated with JSTF showed a dose-dependent increase (Table 2). The latency responses reached a maximum at time interval of 60 min with increase over time, and the pain threshold inhibition was significantly shown at dose of 250 and 500 mg/kg of JSTF as compared to control (*P *< 0.01,* P* < 0.05).

## 4. Discussion

*J. sabina *is a traditional herbal agent which has been used for the treatment of various inflammatory diseases including rheumatism in folk of China. In this study, flavonoids from* J. sabina* (JSTF) have showed the better analgesic and anti-inflammatory effects. Moreover, intragastric administration of grade doses of JSTF gives the maximum dose at 24 g JSTF/kg body, and this finding probably indicates that JSTF is relatively safe in mice.

The metabolic pathways of arachidonic acid (AA) are one of important targets for research and development of new drugs [21]. The onset of inflammatory process is closely associated with AA's metabolites. Under the inflammation stimulation, the phosphatidase is activated, which promote membrane phospholipids metaboliting as AA [22]. The further metabolic pathways of AA have two ways: catalytic metabolized prostaglandin (PGs) by cyclooxygenase (COX) and catalytic generated leukotrienes (LTs) by 5-lipoxygenase (5-LO) [23]. The LTB_4_ in LTs has been closely related to gastrointestinal side effects of nonsteroidal anti-inflammatory drugs (NSAID) [24]. Therefore, the studies of drugs with simultaneously inhibiting effects on COX and LO have vital significance for treatment of inflammatory. COX-1 is a constitutive enzyme of maintaining the body's normal physiological function, while the expression of COX-2 can be significantly increased after simulation by inflammatory factors [25]. JSTF could significantly inhibit the activities of COX-2 and 5-LO, and this extract could play an anti-inflammatory role by influencing metabolic pathways of AA.

The inflammation can be divided into three types including infectious, nonspecific, and allergic inflammation according to its pathogenesis [26]. The studies of anti-inflammatory drugs are usually used by nonspecific animal models as follows: the carrageenan, egg albumin or histamine-induced rats paw edema, xylene-induced ear edema in mice, and capillary permeability in mice as well as cotton pellet granuloma in rats [27]. Thereinto, the cotton pellet granuloma in rats is an excellent chronic inflammatory model that was selected to investigate chronic inflammation which includes a transudative phase and a proliferative phase [28]. Inflammatory response can be readily detected by such formation of granuloma, extravasations, and various biochemical exudates through the cotton pellet granuloma experiment. The dry weight of the implanted cotton pellet correlates well with the amount of granulomatous tissue formation [29]. JSTF could reduce the dry weights of implanted cotton pellets, indicating that it may inhibit the proliferative phases of inflammation.

Xylene-induced ear edema is commonly used acute inflammation model. Our results showed that JSTF can markedly inhibit the formation of xylene-induced ear edema. Ear edema may involve release of inflammatory mediators of promoting vasodilation and increasing vascular permeability, and these mediators can induce ear edema. JSTF may produce the anti-inflammation effect by influencing the actions of the above mediators. In the acetic acid induced vascular permeability in mice, acetic acid causes the increase of some inflammatory substances (prostaglandin E2 (PGE2), prostaglandin F2 (PGF2), serotonin, and histamine) in peritoneal fluids, and these substances may lead to a dilation of the capillary vessels and the increase in vascular permeability. JSTF could markedly inhibit the acetic acid-induced increase in vascular permeability in mice. This result further suggests that JSTF may produce anti-inflammatory effects through inhibiting the inflammatory mediators of the acute phase of inflammation.

Carrageenan-induced inflammation in rats includes two different phases: the initial phase has been involved the release of histamine, serotonin, and bradykinin on vascular permeability; the later phase has been due to over production of prostaglandin in tissues. Namely, development of edema with carrageenan is a biphasic model which involves the contribution of vascular and inflammatory mediators. Initial phase (0-1 h) of edema is attributed by the release of histamine, 5-hydroxytryptamine, and bradykinin. During the 2nd phase (1–5 h) of edema development elevated level of prostaglandins. On the other hand, egg albumin-induced oedema results from the release of histamine and serotonin. In this study, JSTF showed significant inhibitory effect on rat paw oedema development in the middle phase and more pronouncedly in the later phase of carrageenan and egg albumin-induced inflammation. This suggests that JSTF possibly acts by inhibiting the release and/or actions of vasoactive substances (histamine, serotonin, and kinins) and prostaglandins. Moreover, histamine may induce paw edema in rats by evoking the release of prostaglandins and inflammatory mediators. JSTF may act on the inflammatory mediators and inhibit the release of prostaglandins and histamine mediators which causes mucus secretion and mucosal edema.

Acetic acid-induced abdominal writhing response, hot-plate test, and formalin-induced licking response are commonly useful models for evaluation of antinociceptive drugs [30]. The first model is chemical induced nociception for detecting both central and peripheral analgesics. The prostaglandins (PG) in peritoneal exudates are significantly increased after acetic acid by intraperitoneal injection, and PG could induce the abdominal constrictions in mice which are related with sensitization of nociceptors to prostaglandins [31]. The second model (nociception induced by thermal) is mainly undertaken to investigate if drugs have any central analgesic effect [32]. Formalin-induced licking response in mice can be used to screen weak analgesics agents. The pain induced by formalin was long-lasting and divided into two phases as follows: first phase (0-5 min), formaldehyde directly stimulates nerve endings; second phase (20-40 min), inflammatory mediators are produced and released [33, 34]. JSTF could significantly cause a significant dose-dependent inhibition on acetic acid induced writhing response in mice and produce time-course increase in pain threshold in hot-plate test also. Furthermore, pretreatment with JSTF could significantly ameliorate formalin-induced pain in mice in the late phase on dose-dependent way.

The antinociceptive and anti-inflammatory activities of JSTF may be from the synergic effects of its components such as rutin, quercitrin, and isoquercitrin. Antinociceptive and anti-inflammatory effective of these compounds have been reported by some references. For example, quercitrin could significantly ameliorate acetic acid increased vascular permeability in mice [35] and significantly decreased the number of writhing in mice [36]. Quercitrin (50, 100, and 200 mg/kg) inhibited the rat hind paw edema induced by various phlogistics (carrageenin, dextran, histamine, serotonin, and bradykinin) in a dose-dependent manner, and 200 mg/kg of this compound also inhibited the scald edema induced by hot water [37]. Quercitrin has also significantly inhibiting effect on acetic acid induced visceral pain in mice, with a mean IC_50_ value of 2.4 mg/kg [38]. Rutin (100, 200 mg/kg) showed inhibiting effective on Freund's adjuvant arthritis in rats and could improve kidney function and protection of immune organs including spleen and thymus [39]. Moreover, isoquercitrin possesses anti-inflammatory effective also, and its mechanism may be related to inhibition the expression of TNF-*α*, NO, iNOS, and COX-2 [40].

## 5. Conclusion

Traditional medicines as natural therapeutic remedies have been used in all over the world for thousands of years, and it is widely accepted that multiple constituents are responsible for their efficacy. This experimental result indicated that JSTF has analgesic and anti-inflammatory effects, and usage is safe. These pharmacological activities provide pharmacological evidence for the folk use of for treatment of* J. sabina *also. Further molecular and cellular experiments will be carried out to explore its effective mechanisms of JSTF including its active components.

## Figures and Tables

**Table 1:** Effects of JSTF on the granuloma with cotton pellet in rats.

Group	Dose (mg/kg)	Granuloma wt (g)	Inhibiton (%)
Control	—	0.1807±0.0112	—
Aspirin	200	0.1028±0.0271*∗*	43.11
JSTF	500	0.1409±0.0202*∗*	22.02
	250	0.1473±0.0182*∗*	18.48
	125	0.1529±0.0148*∗*	15.38

Mean ± S.D., n=10; *∗P*<0.01, compared with normal control group.

**Table 2:** Inhibition of pain threshold in hot plate test of mice by different treatment.

Group	Dose (mg/kg)	before treatment	After treatment			
			30 min	60 min	90 min	120 min
Control	—	16.85±1.368	17.25±1.849	17.16±1.864	17.35±1.630	19.03±2.793
Aspirin	200	17.05±1.886	26.14±3.051*∗*	32.02±9.539*∗*	29.57±3.093*∗*	27.27±2.579*∗*
JSTF	500	16.97±2.593	23.01±2.209*∗*	29.28±5.245*∗*	25.58±5.597*∗*	24.33±3.532^#^
	250	16.94±2.002	21.20±2.631*∗*	25.49±10.94^#^	23.71±1.978*∗*	22.90±2.674*∗*
	125	16.74±2.612	19.87±2.483^#^	23.57±4.158^#^	21.11±2.402^#^	19.17±2.179

Mean ± S.D., n=10; *∗P*<0.05, *∗∗P*<0.01, compared with normal control group.

## Data Availability

The data used to support the findings of this study are available from the corresponding author upon request.
